# Bipolar Disorder Psychosis Risk Predicts Cue Discrimination on the AX‐Continuous Performance Task Paradigm

**DOI:** 10.1111/bdi.70129

**Published:** 2026-06-09

**Authors:** Meghan Fiske, Dominique L. DiDomenico, Henry W. Chase, Yvette Afriyie‐Agyemang, Genna Bebko, Haris A. Aslam, Simona Graur, Osasumwen Benjamin, Yiming Wang, Richelle Stiffler, Michele Bertocci, Brian A. Coffman, Deepak K. Sarpal, Cameron S. Carter, Mary L. Phillips

**Affiliations:** ^1^ Department of Psychiatry University of Pittsburgh School of Medicine Pittsburgh Pennsylvania USA; ^2^ Department of Psychiatry and Human Behavior University of California, Davis Sacramento California USA

**Keywords:** AX‐CPT, bipolar disorder, mania affective lability, psychosis, sustained attention

## Abstract

**Objectives:**

Bipolar Disorder (BD)—characterized by mania, affective lability, and elevated psychosis risk—is associated with sustained attention deficits. However, evidence regarding performance on the AX‐Continuous Performance test task (AX‐CPT), a task reliably impaired in schizophrenia‐spectrum psychosis, is mixed. This study examined whether individual differences in mania/affective lability risk and psychosis risk, across cohorts of BD and BD‐at‐risk individuals, were associated with altered AX‐CPT performance.

**Methods:**

The standard measures d'context and A‐cue bias quantified AX‐CPT performance. Factors from the Mood Spectrum Self Report (MOODS‐SR‐L) indexed lifetime mania/affective lability risk (psychomotor activation, suicidality, and mixed instability) and lifetime psychosis risk. Linear regressions were performed for dimensional continuous aims (Aims 1–2) for both d'context and A‐cue bias. Tests between BD with low and high‐risk groups (Aims 3–4) were completed with ANCOVAs for both AX‐CPT output measures.

**Results:**

In the final sample of euthymic BD (*n* = 27) and at‐risk individuals (*n* = 121), higher levels of psychosis and mania/affective lability risk were associated with reduced target discrimination (d'context; absolute *t*'s > 2.015, *p*'s < 0.047), but not with altered A‐cue bias. Group comparisons showed no significant differences for either AX‐CPT measure. Associations with d'context from Aims 1–2 remained after covarying for current depressive symptoms but were removed when covarying for current mania severity (absolute *t*'s < 1.26, all *p*'s > 0.203).

**Conclusions:**

Target discrimination deficits were associated dimensionally with psychosis and mania/affective lability risk but not categorically with BD diagnosis. This suggests scope for dimensional risk models for understanding sustained attention deficits in BD and at‐risk individuals and highlights contributions of mania/affective lability and psychosis risk.

## 1 Introduction

Bipolar disorder (BD) is a chronic and deleterious psychiatric illness characterized by recurrent mood episodes, with a considerable risk of suicide attempt [1, 2, 3]. The defining characteristic of BD is mania/hypomania and the associated affective lability, which includes elevated impulsivity, marked by rapid, unplanned reactions to stimuli without consideration of consequences [4, 5, 6]. A significant number of individuals with BD also experience comorbid psychosis, with the prevalence of psychotic symptoms in BD‐1 estimated to be as high as 54% [7]. Greater severity of mania/hypomania and psychotic symptoms in BD are associated with worse clinical and psychosocial outcomes overall [8, 9, 10]. It is therefore imperative to identify behavioral markers that detect individuals at elevated risk of mania and psychosis, and to develop target interventions.

Within BD, mania/hypomania and psychotic symptoms are associated with a wide range of cognitive impairments, including difficulties with verbal fluency, working memory, problem solving, and cognitive flexibility [11, 12, 13, 14, 15]. Among cognitive domains affected in BD, sustained attention, the ability to remain responsive to repetitive cues over an extended period, has been consistently impaired [16, 17, 18, 19]. Furthermore, sustained attention deficits tend to worsen with increased frequency of prior manic episodes [20]. Meanwhile, such deficits appear relatively stable and are apparent regardless of mood state, suggesting that they may serve as a trait marker [21, 22, 23]. Despite the thorough documentation of sustained attention impairment in BD, it remains unclear whether impairments are evident at illness onset or in individuals at future risk of BD. While there is evidence that sustained attention deficits are a stable vulnerability marker for individuals at high risk of schizophrenia, including unaffected first‐degree relatives [24], the extent to which these deficits are observed in individuals at future risk of BD remains unknown.

One task that has demonstrated utility in examining sustained attention, especially within schizophrenia, is the AX‐Continuous Performance Task (AX‐CPT), designed to assess the maintenance and use of contextual cues to optimize attentional performance [25]. Key performance metrics include the d'context, which measures the use of contextual information to discriminate between target and nontarget responses, and the A‐cue bias, which assesses biased utilization of a predictive cue to guide responses to the target. Both measures are informed by signal detection theory [26]: in particular, the capacity to discriminate between targets and a response bias elicited by predictive cues. While d'context is reliably reduced in individuals with schizophrenia [18, 27, 28], there are few and mixed findings within the BD literature, with some studies showing intermediate deficits in relation to schizophrenia and others showing no alteration in d'context relative to healthy controls [16, 29, 30, 31, 32]. Furthermore, there are discrepant findings regarding the A‐cue bias in individuals with BD. While some studies suggest an increase in cue utilization under reward conditions in individuals with BD [33], others suggest that cue utilization is not altered [16, 34, 35]. By contrast, predictive learning impairments can be observed in BD, which may reduce the capacity of the A‐cue to bias target responses [36].

Importantly, most research utilizing the AX‐CPT compares individuals with BD, individuals with schizophrenia, and healthy control participants. Although few studies examined performance on the AX‐CPT with psychosis risk [18, 32], no studies to our knowledge examined individuals at risk for BD with subthreshold or threshold mania and mood dysregulation symptoms. One scale that can be used to measure the magnitude of BD risk dimensionally is the Mood Spectrum Self Report (MOODS‐SR‐L), designed to measure lifetime depression and mania/hypomania risk [37]. This is a self‐report instrument designed to assess a comprehensive array of BD‐related symptoms across an individual's lifetime, acute changes in clinical status, and to accurately measure an individual's risk for development of mood disorders [38]. We selected several factors within the instrument to evaluate individual differences in BD psychosis and mania/affective lability risk. The psychotic features factor quantifies predisposition to psychosis, and three factors (psychomotor activation, mixed instability, and suicidality) reflect a predisposition to mania/affective lability risk. Furthermore, these three factors are most effective in discriminating between Major Depressive Disorder and BD [39].

In the present study, our overarching goal was to determine the extent to which mania/affective lability risk and psychosis risk were associated with altered performance on the AX‐CPT. Our aims were twofold: first, we adopted a dimensional, continuous approach to identify (1) psychosis and (2) mania/affective lability risk‐specific sustained attention deficits. Then, we determined the extent to which task performance differed among individuals with BD versus those at low and high psychosis risk (3) and low and high mania/affective lability risk (4). Our specific hypotheses were: (1) a greater predisposition to psychosis would be associated with a lower d'context, similar to the deficit observed in individuals with/at risk for schizophrenia [18, 31, 32]; (2) greater mania/affective lability risk would also be associated with lower d'context [16, 30]; (3) individuals with BD would show a lower d'context compared with those at low risk, but not high risk, for psychosis; (4) individuals with BD would show a significantly lower d'context compared with those at low risk, but not high risk, for mania/affective lability. The paucity of extant data prevented more nuanced hypotheses regarding psychosis and mania/affective lability risk associations with A‐cue bias and between group differences in A‐cue bias.

## 2 Methods

### 2.1 Participants

The at‐risk group of participants was recruited from the University of Pittsburgh and the surrounding community through student counseling centers, participant registries, and community advertisements (in the context of a larger study, R37MH100041[PI:Phillips]). A transdiagnostic approach allowed recruitment of participants across a wide range of disorders that predispose to future BD. The final risk sample (*N* = 121) consisted of adults aged 18–30 years (M = 23.44, SD = 3.35) with disorders other than BD as determined by the SCID5‐RV [40] (*n =* 46), or healthy control (*n* = 75). The 46 individuals with other diagnoses consisted of Major Depressive Disorder (*n =* 23), Persistent Depressive Disorder (*n =* 2), Premenstrual Depressive Disorder (*n =* 1), Alcohol Substance Use Disorder (*n =* 3), Social Anxiety Disorder (*n =* 2), Posttraumatic Stress Disorder (*n =* 5), Generalized Anxiety Disorder (*n =* 1), Attention‐deficit Hyperactivity Disorder (*n =* 3), Obsessive Compulsive Disorder (*n =* 3), Binge Eating Disorder (*n =* 1), Not otherwise specified Feeding or Eating Disorder (*n =* 1), or an Adjustment Disorder (*n =* 1). The sample was predominantly female (64.5%) and white (63.6%).

In addition, a sample of BD individuals was recruited in euthymic mood state, in order to remove large variance in present symptom severity on dependent variables. The BD sample included BD type I (*n =* 9) [1, 2, 3] and BD type II (*n =* 18), given that these subtypes have been most frequently examined in studies focusing on neurobiological and neurocognitive alterations in BD [41]. The final sample (*N* = 27), including adults from ages 18–30 years (M = 24.56, SD = 3.67), was predominantly female (59.3%) and white (81.5%). Of the 27 BD participants, 23 were taking psychotropic medications.

Participants in the at‐risk and BD groups did not significantly differ on age, sex at birth, IQ, or years of education. See Tables 1 and 2 for more details concerning demographic information.

**TABLE 1:** Clinical and demographic data.

	At risk	BD	Statistics	*p*
	(*n* = 121)	(*n* = 27)		
Age (years)	23.44 (3.35)	24.56 (3.66)	*t*(36.32) = 1.45	0.154
Sex at birth (female)	82	21	*χ* ^2^ = 1.05	0.307
IQ (NAART)	110.34 (6.95)	111.56 (5.24)	*t*(146) = 0.86	0.392
Education			*χ* ^2^ = 4.23	0.517
Partial High school	1	0		
High school Diploma or GED	14	1		
Some College	39	11		
Technical school or Associates	2	0		
College Diploma	46	8		
Graduate or Professional Degree	19	7		
Clinical measures				
Mania (YMRS)	0.41 (0.92)	1.30 (1.33)	*t*(31.8) = 3.29	0.001
Depression (HAMD)	2.42 (4.10)	4.78 (4.26)	*t*(146) = 2.62	0.004
Anxiety (HAMA)	1.76 (3.20)	3.67 (3.03)	*t*(146) = 2.82	0.003

Abbreviations: %, percentage of subsample size; *n*, subsample size.

**TABLE 2:** Participant medication use by cohort.

Medication use	At risk		BD		Total	
	*n*	%	*n*	%	*n*	%
Antidepressant	1	0.8	12	44.4	13	8.8
Mood stabilizer	0	0.0	20	74.1	20	13.5
Antipsychotic	0	0.0	8	29.6	8	0.5
None	120	99.2	3	11.0	123	83.1

Abbreviations: %, percentage of subsample size; *n*, subsample size.

All participants provided signed informed consent, and the study was approved by the University of Pittsburgh Human Research Protection Office. Diagnostic assessments were conducted using the Structured Clinical Interview for DSM‐5 Research Version (SCID‐5‐RV).

### 2.2 Inclusion/Exclusion Criteria

All participants met the following criteria to be eligible for the study: no history of head injury or neurological disorders; no pervasive developmental disorder; no history of systemic medical disease and treatment; premorbid NAART IQ estimate > 85; no visual disturbance: > 20/40 Snellen visual acuity; right handedness; no history of alcohol/substance use disorder (SUD) and/or illicit substance use (except cannabis at non‐SUD levels, given its common usage in young adults) over the last 3 months determined by SCID‐5‐RV; ability to understand English; no personal history of psychotic disorders, as the larger study (R37MH10004) focuses on BD risk in individuals without a previous history of psychotic disorders, in order to avoid confounds of previous psychotic disorders on measures of interest. Of the 232 participants enrolled, 84 participants were removed from this analysis for not meeting the following criteria: non‐completion of the AX‐CPT (*n = 77*), or a high number of A/B cue omission errors (> 13: all included participants achieved at least 90% accuracy) on the AX‐CPT, suggestive of a lack of understanding of the task (*n = 7*).

The final sample size (*N* = 148) is adequately powered to detect effect sizes comparable to current literature regarding the AX‐CPT and risk for psychosis spectrum disorders, with small to medium effect sizes (*g* = 0.4) [31].

### 2.3 Measures

#### 2.3.1 Clinical Assessments

For all participants, the Hamilton Rating Scale for Depression (HAMD) assessed present depression severity [42], the Hamilton Rating Scale for Anxiety (HAMA) assessed present anxiety severity [43], and the Young Mania Rating Scale (YMRS) for present mania/hypomania severity [44].

The psychotic features factor of the MOODS‐SR‐L is a measure of lifetime history of psychosis risk; questions range from examining excess guilt to auditory hallucinations. The other three factors were selected to measure BD mania/affective lability risk [39]. These three variables of interest (suicidality, psychomotor activation, and mixed instability) were strongly inter‐correlated (all *r*'s > 0.7), suggesting the presence of a single latent variable representing most of the variation and a lack of additional information from different measures. This was confirmed using Principal Component Analysis, which demonstrated that the first principal component explained 83.11% of the variance in the three scales. Therefore, we pooled the three variables into a single factor by *z*‐scoring each variable and determining the mean for each participant.

To stratify risk, we created psychosis risk groups as those at no risk for psychosis, scoring zero (*n* = 57), and at risk endorsing one or more questions within the psychotic factor (*n* = 64). We also created risk groups based on the mean factor score of the mania/affective lability risk single factor, with roughly equally distributed quartiles: low risk (*n =* 37, m = −0.63, stdev = 0.00), low‐medium risk (*n =* 23, m = −0.42, stdev = 0.06), medium‐high risk (*n =* 30, m = −0.13, stdev = 0.18), and high risk (*n =* 31, m = 1.18, stdev = 0.78).

#### 2.3.2 AX‐Continuous Performance Task

**FIGURE 1:**
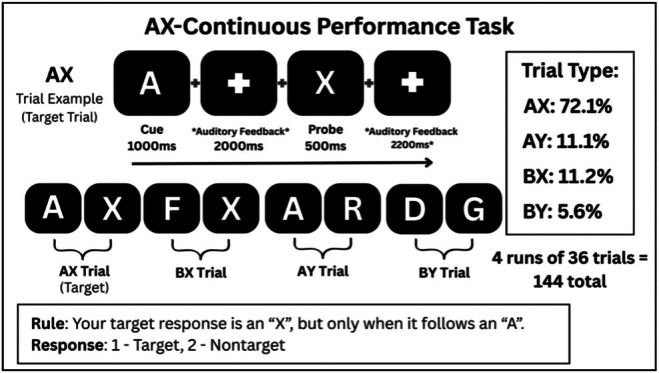
The AX‐continuous performance task trial types and sequence example. Image demonstrating the AX‐CPT paradigm sequence and types.

The AX‐CPT is a computerized test administered in this study on *E‐Prime 2.0* (Psychology Software Tools Inc., 2012; Figure 1) [45], used to measure sustained attention and cognitive control mechanisms. The task requires participants to respond to a probe, contingent upon the preceding cue and consists of four runs of 36 trials. A trial consists of a cue letter followed by a probe letter that is presented after a delay period. Participants make target and non‐target responses to these continuous streams of letter stimuli on a computer screen. During a trial, participants are presented with a cue (A or any letter other than A, collectively referred to as “B‐cues”) presented in the center of the screen for 1000 ms. A fixation cross during an inter‐stimulus interval of 2000 ms follows, which includes auditory feedback (correct/incorrect) for the cue response. Then, the probe (X or any letter other than X, collectively referred to as “Y‐probes”) appears in the center of the screen for 500 ms. Participants are instructed to respond to the letter X probe when it follows an A cue by hitting the “1” key with their pointer finger; this is known as the target response. All other stimuli require a non‐target response; the participant is instructed to hit the “2” key with their middle finger. The proportion of trial types is as follows: a disproportionate 72.2% occurrence of trial type AX (target sequence), 11.1% trial type AY, 11.1% trial type BX, and 5.6% trial type BY. The trials were presented in a randomized order. The high proportion of AX trials conditioned participants to make a prepotent response to the X probe. Thus, the non‐target trial in which a B cue was presented followed by the letter X requires the most cognitive control. Finally, auditory feedback is also provided following the probe, within an inter‐trial interval of 2200 ms.

#### 2.3.3 Outcome Measures (AX‐CPT)

Error rates and response times are recorded for each trial type (AX, AY, BX, BY). Error rates are calculated as (errors + 0.5)/(number of trials + 1) to correct for error rates equal to zero [46]. The computed variables of interest regularly calculated are: d'context, a measure of discrimination of the cue, and A‐cue bias, a measure of sensitivity to the cue. The d'context variable is computed as: z(AXHits)–z(BX False Alarms [FA]), with z representing the z‐transform of the value [25]. This measure, a proportion of trials context is used to derive a target response relative to trials they failed to use context, reflects the participant's sensitivity of context and how well a previous stimulus is detected. In other words, this measure reflects the tendency to make a recency error via responding to the X regardless of the letter seen initially. The A‐cue bias is calculated as: 0.5(z(AXHits) + z(AYFA)). This is a measure of cue utilization, with values of greater magnitude indicating an inability to inhibit the prepotent response, that is, the tendency to respond based upon the A cue alone regardless of the identity of the probe [47].

### 2.4 Statistical Analysis

Data were analyzed using SPSS (Version: 29.0.0.0). See Table 3 for statistics regarding error rates and reaction times.

**TABLE 3:** Descriptive statistics for the AX‐CPT as a function of task condition.

Dependent variable	Trial type	BD		At risk	
		m	stdev	m	stdev
d'context		3.095	0.814	3.181	0.848
A‐cue bias		0.277	0.251	0.201	0.249
Average Error Rates	AX	0.035	0.038	0.038	0.039
	AY	0.102	0.122	0.063	0.081
	BX	0.117	0.107	0.103	0.128
	BY	0.024	0.051	0.020	0.058
Average RT (msec)	AX	408.887	45.608	409.402	55.231
	AY	495.878	47.002	505.164	57.446
	BX	422.927	115.410	421.782	99.210
	BY	404.876	84.734	402.843	79.401

Abbreviations: m, subsample mean; RT, reaction Time for all valid trials (i.e., probe RT following correct cue response); stdev, standard deviation.

Aim 1: Two linear regressions were conducted to examine the associations of psychosis risk with d'context and with A‐cue bias. We completed this analysis with the full sample (*N =* 148) including all BD at‐risk individuals and individuals with BD, with age, sex at birth, and IQ as covariates.

Aim 2: Two linear regressions were conducted within the above participants to examine the associations of mania/affective lability risk with d'context and with A‐cue bias. As a post hoc analysis, to further characterize mania/affective lability risk‐related measures of AX‐CPT performance, we ran three linear regressions: one with each factor (psychomotor activation, mixed instability, and suicidality) as the independent variable and the d'context and A‐cue bias as the dependent measures, with age, sex at birth, and IQ as covariates. To account for multiple tests within this second aim, we applied a Bonferroni adjustment (*α*_(new)_ = 0.05/3 = 0.017).

Aim 3: AX‐CPT performance was compared between participants at no risk for psychosis and those with BD, and between participants at high psychosis risk and those with BD, to further characterize group‐level differences in cognitive control. We conducted four ANCOVAs: first comparing the BD group with the low psychosis risk group on d'context and A‐cue bias; then comparing the BD group with the high psychosis risk group on the same dependent measures, covarying for age, sex, and IQ.

Aim 4: AX‐CPT performance was compared between participants at low mania/affective lability risk and those with BD, as well as between participants at high mania/affective lability risk and those with BD. We conducted four ANCOVAs: first comparing the BD group with the low mania/affective lability risk group on d'context and A‐cue bias; then comparing the BD group with the high mania/affective lability risk group on the same dependent measures, covarying for age, sex, and IQ.

Follow‐up analyses were performed to examine the impact of present affective symptom severity (e.g., HAMD, YMRS) and the impact of medication in individuals with BD on dependent variables. For the former, we included these measures as covariates in regression models when testing hypotheses relating to Aims 1 and 2. For the latter, we divided individuals with BD into medicated and unmedicated groups for each medication class and compared d'context and A‐cue bias between these groups.

## 3 Results

### 3.1 Hypothesis Testing

**FIGURE 2:**
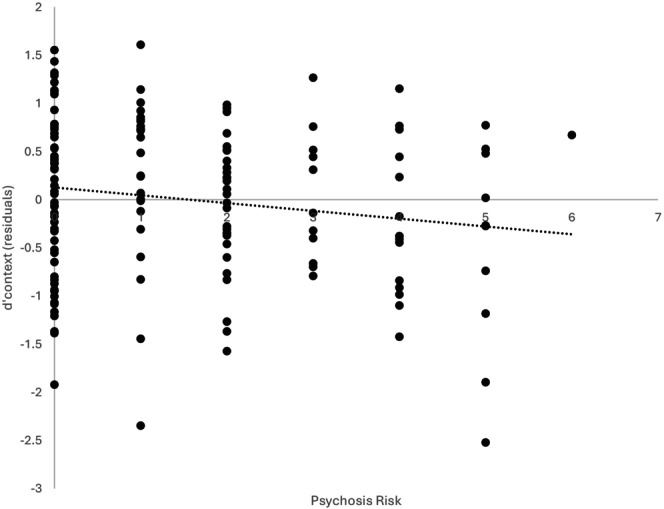
Relationship of d'context with psychosis risk. d’context measure residualized by modeling/removing effects of IQ, sex and age.

Hypothesis (1) Higher levels of psychosis risk was associated with a lower d'context (*B* = −0.083, *t* = −2.015, *p* = 0.046: Figure 2). IQ was associated with d'context (*B* = 0.026, *t* = 2.51, *p* = 0.013), all other covariates were not significantly associated (*p*'s > 0.1). There was no significant relationship between psychosis risk and the A‐cue bias (*B* = 0.001, *t* = 0.095, *p* = 0.925).

**FIGURE 3:**
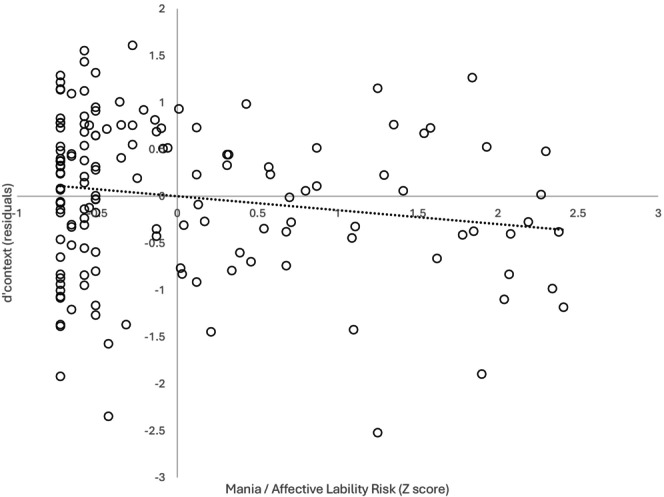
Relationship of d'context with mania/affective lability risk. d'context measure residualized by removing effects of IQ, sex, and age.

Hypothesis (2) Higher mania/affective lability risk was associated with a lower d'context (*B* = −0.150, *t* = −2.02, *p* = 0.045: Figure 3), but not significantly associated with A‐cue bias (*B* = 0.009, *t* = 0.375, *p* = 0.708). Within the individual selected factors, there was a significant relationship between the d'context and mixed instability (*B* = −0.078, *t* = −2.51, *p* = 0.013), a trend level relationship with suicidality (*B* = −0.076, *t* = −1.99, *p* = 0.048), but no relationship with psychomotor activation (*B* = −0.016, *t* = −1.039, *p* = 0.301). After applying a Bonferroni correction for these three tests (*α*_(new)_ = 0.05/3 = 0.017), only mixed instability was significant. With the three selected factors, there were no relationships between the A‐cue bias and either mixed instability (*B* = 0.0005, *t* = 0.005, *p* = 0.996), suicidality (*B* = 0.005, *t* = 0.457, *p* = 0.649), or psychomotor activation (*B* = 0.003, *t* = 0.566, *p* = 0.572). The covariate IQ was a significant predictor of d'context in the regression models for overall mania/affective lability risk, suicidality, mixed instability, and psychomotor activation (all *p*'s < 0.013).

Hypothesis (3) The no‐psychosis risk group and the BD group did not significantly differ on d'context (*F*(1,79) = 0.691, *p* = 0.408) or A‐cue bias (*F*(1,79) = 2.75, *p* = 0.101). The psychosis risk group and the BD group did not significantly differ on d'context (*F*(1,86) = 0.721, *p* = 0.398) or A‐cue bias (*F*(1,86) = 0.234, *p* = 0.630).

Hypothesis (4) The low mania/affective lability risk group and the BD group did not significantly differ on d'context (*F*(1,59) = 0.312, *p* = 0.579), although sex was a significant covariate (*F*(1,59) = 4.66, *p* = 0.035), with male participants having greater target discrimination deficits. The low mania/affective lability risk group and BD group did not significantly differ on A‐cue bias (*F*(1,59) = 1.33, *p* = 0.254). The high mania/affective lability risk group and the BD group did not significantly differ on d'context (*F*(1,53) = 0.035, *p* = 0.853) or A‐cue bias (*F*(1,53) = 1.81, *p* = 0.184).

### 3.2 Follow Up Analyses: Illness Severity

We then examined the impact of including HAMD and YMRS to control for present affective symptom severity in regression models from Aims 1 and 2. HAMD scores were not significantly associated with either d'context (*B* = 0.004, *t* = 0.246, *p* = 0.806) or A‐cue bias (*B* = 0.007, *t* = 1.45, *p* = 0.149). Similarly, YMRS scores were not significantly associated with d'context (*B* = −0.095, *t* = −1.45, *p* = 0.148) or A‐cue bias (*B* = 0.013, *t* = 0.661, *p* = 0.509). When covarying for HAMD scores, the psychosis risk score (*B* = −0.097, *t* = −2.04, *p* = 0.043), the mania/affective lability risk score (*B* = −0.163, *t* = −1.99, *p* = 0.048), and the mixed instability factor (*B* = −0.081, *t* = −2.48, *p* = 0.014) remained significant predictors of d'context. Including YMRS score as a covariate, however, removed the significance of the relationships between psychosis risk (*B* = −0.06, *t* = −1.28, *p* = 0.203), mania/affective lability risk (*B* = −0.112, *t* = −1.26, *p* = 0.209), and mixed instability (*B* = −0.066, *t* = −1.90, *p* = 0.06) with d'context. To address relationships between the clinical variables (HAMD, YMRS, and MOODS_SR_L factors), we determined the inter‐relationships among all variables using Spearman's Rho (Table 4). These showed numerically stronger associations between the MOODS SR factors and YMRS than with HAMD, consistent with the statistical effect of including YMRS in the d'context regression models.

**TABLE 4:** Associations among current illness severity measures.

	MOODS psychotic symptoms	MOODS mania/affective lability	MOODS suicidality	MOODS psychomotor activation	MOODS mixed instability
YMRS	0.549	0.552	0.569	0.553	0.417
HAMD	0.537	0.485	0.501	0.440	0.413

*Note:* Associations described with Spearman's rho (all *p*'s < 0.001).

No significant effect of medication was observed (see Supporting Information).

## 4 Discussion

In the present study, we sought to evaluate whether AX‐CPT performance was related to individual differences in psychosis and mania/affective lability risk in groups of individuals with BD and individuals at an elevated risk of mood disorders. The AX‐CPT is an established assay of cognitive function in schizophrenia, associated with medium/large effect sizes relative to healthy control participants. For both psychosis and mania/affective lability, increasing levels of risk—as determined by the MOODS‐SR‐L scale—were associated with reduced target discrimination (d'context) but not significantly associated with A‐cue bias. Furthermore, categorical analyses conducted separately for BD versus low mania/affective lability risk and BD versus low psychosis risk showed no between‐group differences on these indices of task performance. Together, these findings suggest that the reduction in d'context, often reported in patients with schizophrenia/psychotic spectrum disorders [31], is present in both individuals at an elevated risk for psychosis and mania/affective lability. Furthermore, both psychosis risk and mania/affective lability risk reductions in target discrimination were related to present mania, but not depression, severity. Thus, impaired AX‐CPT performance in subthreshold individuals might indicate shared deficits for these dimensions of psychopathology, with these dimensions associated more with present mania than present depression severity.

There was a reduction in the d'context variable with increasing psychosis risk and increasing mania/affective lability risk. This finding suggests a shared sustained attention deficit, which may manifest as difficulty maintaining and integrating context to guide responses. Of the three subscales used to define mania/affective lability risk, the strongest relationship with d'context was observed with the mixed instability subscale, which reached corrected significance. Suicidality was associated at trend level, followed by psychomotor activation. The reduction of d'context associated with the mixed instability factor– comprising questions concerning disinhibition, impulsivity, and sensation seeking– suggests that rapid affective shifts and behavioral reactivity tax sustained attention resources necessary for context maintenance. This finding indicates that aspects of BD symptomatology, with mixed instability showing the strongest relationship, are similarly relevant for d'context deficits as psychosis risk. The two sets of risk scores were strongly related across individuals, which makes delineating independent contributions of different dimensions difficult. The findings are consistent, however, with the complex and close overlap between psychosis and BD [48]. Thus, there may be a dimension underlying shared symptomatology across schizophrenia, characterized by underlying risk for psychosis and BD, characterized by underlying risk for both mania/affective lability and psychosis.

While deficits in target discrimination were not directly associated with current mania (YMRS) or depression (HAMD) severity, covarying for mania severity eliminated the previously significant relationships between d'context and both mania/affective lability risk and psychosis risk, whereas covarying for depression severity did not alter these associations. This finding suggests that BD‐associated sustained attention deficits are linked with mania rather than depression. Consistent with these findings, previous research indicates that mania is associated with impairments in attention [49] goal maintenance, and distractibility [12], while depression in BD has been associated with psychomotor slowing and diminished processing efficiency [50].

No significant association was detected with the A‐cue bias and psychosis risk or mania/affective lability risk within this sample. The absence of significant effects for A‐cue bias suggests that response tendencies (expecting targets after A cues) might remain intact even when target discrimination is diminished. Prior research suggests that cue utilization might be altered within BD samples under specific reward conditions [33]; thus, the absence of reward manipulations in the current study might partially explain our null findings regarding A‐cue bias. Additionally, the findings with d'context and not the A‐cue bias may be related to their differential sensitivity to cognitive control demands elicited by the prepotent tendency to give a target response to the X probe. Thus, the relative increase in difficulty for BX trials rather than AY trials may explain the absence of our findings regarding the A‐cue in this sample.

Group‐level comparisons showed no significant differences in either d'context or A‐cue bias. Specifically, the low risk for psychosis or mania/affective lability groups did not significantly differ from individuals with BD in d'context or A‐cue bias. Our findings therefore suggest that reductions in d'context reflect dimensions of psychopathology rather than diagnostic categories with regards to psychosis and mania/affective lability risk. Previous BD studies have typically employed a categorical approach, evaluating performance across diagnostic groups. While few papers have examined AX‐CPT performance and current symptom severity across the course of mood disorders [16, 21] no studies to our knowledge examined relationships among AX‐CPT performance and individual differences in risk for mood disorders. This two‐pronged approach, dimensional and categorical, has several notable strengths. First, the continuous approach aligns with the NIMH Research Domain Criteria [51], which conceptualizes psychiatric illness as dimensions of psychopathology. Secondly, the categorical approach reflects much of current research with the AX‐CPT and schizophrenia research [19, 25, 27, 52]. By adopting both approaches, we therefore aimed to draw direct comparisons between prior research with schizophrenia and schizophrenia risk and mania/affective lability risk and BD (Aim 4). Overall, the dimensional approach revealed more about sustained attention deficits associated with BD and BD risk than the categorical approach.

The magnitude of the resulting effect sizes between d'context and psychosis or mania/affective lability risk scores were smaller than those obtained with case‐controlled designs with schizophrenia. The upper limit of the 95% confidence intervals around our estimated effect sizes reached around Cohen's *d* = 0.37, smaller than the effect sizes of *d* > 0.8 reported in a meta‐analysis of schizophrenia [31]. If the dimension selected for individual differences analysis aligns with the underlying psychopathology, the individual differences approach should, however, be more sensitive, a priori, than the case‐controlled design [53]. An explanation is that our dimensional approach is indeed more sensitive, but that overall symptom severity was much lower in our sample than in individuals with schizophrenia. Notably, the dimensional approach to explaining differences in AX‐CPT performance has been somewhat less successful in schizophrenia, with modest evidence for a relationship with disorganization symptoms and non‐significant relationships of positive and negative symptoms [31]. The relationship between d'context and psychosis risk in the context of BD and BD risk might therefore follow a non‐linear function, in which there are dimensional relationships in currently nonpsychotic BD at risk individuals and individuals with BD, but a much tighter coupling of d'context and psychotic symptom severity at the onset of psychosis.

There were limitations to the present study. The small BD sample size (*n* = 27) may have limited power to detect group‐level differences, although it was adequately powered to evaluate effects of the magnitude of those seen in schizophrenia. Most individuals with BD were medicated and euthymic, although our follow up analyses revealed no significant relationships with medication classes, and research indicates that BD individuals display cognitive impairments even when euthymic [54]. There were significant positive associations between IQ and d'context, suggesting IQ might serve as a protective factor among at‐risk individuals and those who develop BD, but this relationship did not obscure the significant findings regarding the impact of mania/affective lability and psychosis risk on sustained attention. Similarly, there was an effect of sex, with male participants having a lower d'context than female participants, but only when examining between group difference in d'context in Aim 4. Although research on the AX‐CPT in schizophrenia has not found associations with sex [55], studies examining cognitive deficits in BD reveal sex differences in attention tasks, with male participants performing worse than female participants [56].

## 5 Conclusions

Our findings help to characterize cognitive dysfunction across the spectrum of those at risk for and those with BD. They suggest that a dimensional approach, based on self‐report measures of psychosis and mania/affective lability risk, is more insightful than a diagnostic, categorical approach in elucidating the nature of underlying cognitive deficits in BD. Future studies can aim to replicate findings in independent samples including psychotic individuals with BD and/or individuals with schizoaffective disorder in order to confirm and expand upon these findings.

## Funding

This work was supported by the National Institute of Mental Health (R37MH100041).

## Conflicts of Interest

The authors declare no conflicts of interest.

## Supporting information


**Table S1:** Medication Types as Predictors of AX‐CPT variables within BD group.

## Data Availability

All clinical data for the analyses reported in the present study can be found at: https://nda.nih.gov/edit_collection.html?id=3397. The AX‐CPT output data is available upon request.
