# Supplementary material for: Bipolar Disorder Psychosis Risk Predicts Cue Discrimination on the AX‐Continuous Performance Task Paradigm
Source: Bipolar Disord. 2026 Jun 9;28(4):e70129. doi: 10.1111/bdi.70129 (PMC13248585; doi:10.1111/bdi.70129)
Supplement: Supplementary file 1 — Table S1: Medication Types as Predictors of AX‐CPT variables within BD group. [file BDI-28-0-s001.docx]

**Bipolar Disorder Psychosis Risk Predicts Cue Discrimination Performance on the AX-CPT Paradigm**

**Supplementary Information**

**Medication effects**

Within the BD sample, 23 participants were taking either antidepressants (*n*=12), mood stabilizers (*n*=20), antipsychotics (*n*=8), or a combination of these medications. To evaluate the influence of psychotropic medication on sustained attention, six linear regression models with d’context (1) and A-cue bias (2) as the dependent variables, and each of the three medication classes as predictors were run. Age, sex, and IQ were included as covariates. Within the BD group, there was no significant effect of medication class on either d’context or A-cue bias (all *p*’s>.34). See Table S1.

Additionally, we sequentially excluded all individuals reporting use of each medication class and performed the same ANCOVAs used in Aims 3 and 4 as medication use could have confounded group effects. These analyses examined whether group differences in A-cue bias or d’context persisted after excluding individuals taking each medication class. When excluding those taking antidepressants (*n*=13), antipsychotics (*n*=8), or mood stabilizers (*n*=13), there were no significant relationships between those at low risk for psychosis, and those with BD on A-cue bias or d’context (all *p*’s>0.1). There were also no between group differences for those at high risk for psychosis and participants with BD in d’context or A-cue bias. When sequentially removing the same participants in each medication class there were no significant relationships between those at low mania/affective lability risk and participants with BD (all *p*’s>0.3). Similarly, there were no significant differences between those at high mania/affective lability risk and participants with BD on A-cue bias or d’context (all *p*’s>0.3).

**Table S1**

*Medication Types as Predictors of AX-CPT variables within BD group*


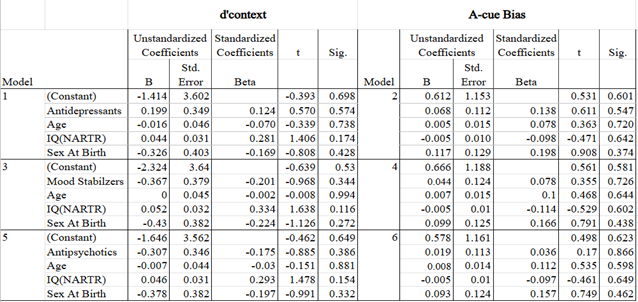


*Note.* *Six individual linear regressions within the BD group (n=27) analyzing medication class and AXCPT output variables (A-cue and d’context)*
